# Prospects and challenges for the conservation of farm animal genomic resources, 2015-2025

**DOI:** 10.3389/fgene.2015.00314

**Published:** 2015-10-21

**Authors:** Michael W. Bruford, Catarina Ginja, Irene Hoffmann, Stéphane Joost, Pablo Orozco-terWengel, Florian J. Alberto, Andreia J. Amaral, Mario Barbato, Filippo Biscarini, Licia Colli, Mafalda Costa, Ino Curik, Solange Duruz, Maja Ferenčaković, Daniel Fischer, Robert Fitak, Linn F. Groeneveld, Stephen J. G. Hall, Olivier Hanotte, Faiz-ul Hassan, Philippe Helsen, Laura Iacolina, Juha Kantanen, Kevin Leempoel, Johannes A. Lenstra, Paolo Ajmone-Marsan, Charles Masembe, Hendrik-Jan Megens, Mara Miele, Markus Neuditschko, Ezequiel L. Nicolazzi, François Pompanon, Jutta Roosen, Natalia Sevane, Anamarija Smetko, Anamaria Štambuk, Ian Streeter, Sylvie Stucki, China Supakorn, Luis Telo Da Gama, Michèle Tixier-Boichard, Daniel Wegmann, Xiangjiang Zhan

**Affiliations:** ^1^School of Biosciences, Cardiff UniversityCardiff, UK; ^2^Sustainable Places Research Institute, Cardiff UniversityCardiff, UK; ^3^Faculdade de Ciências, Centro de Ecologia, Evolução e Alterações Ambientais (CE3C), Universidade de LisboaLisboa, Portugal; ^4^Centro de Investigação em Biodiversidade e Recursos Genéticos (CIBIO-InBIO), Universidade do Porto, Campus Agrário de VairãoPortugal; ^5^Food and Agriculture Organization of the United Nations, Animal Genetic Resources Branch, Animal Production and Health DivisionRome, Italy; ^6^Laboratory of Geographic Information Systems (LASIG), School of Civil and Environmental Engineering (ENAC), Ecole Polytechnique Fédérale de LausanneLausanne, Switzerland; ^7^Laboratoire d'Ecologie Alpine, Université Grenoble AlpesGrenoble, France; ^8^Faculty of Sciences, BioISI- Biosystems and Integrative Sciences Institute, University of LisbonCampo Grande, Portugal; ^9^Parco Tecnologico PadanoLodi, Italy; ^10^BioDNA Centro di Ricerca sulla Biodiversità a sul DNA Antico, Istituto di Zootecnica, Università Cattolica del Sacro Cuore di PiacenzaItaly; ^11^Faculty of Agriculture, University of ZagrebZagreb, Croatia; ^12^Natural Resources Institute Finland (Luke), Green TechnologyJokioinen, Finland; ^13^Institut für PopulationsgenetikVetmeduni, Vienna, Austria; ^14^NordGen -The Nordic Genetic Resource CenterÅs, Norway; ^15^Livestock Diversity Ltd.Lincoln, UK; ^16^School of Life Sciences, University of NottinghamNottingham, UK; ^17^Department of Animal Breeding and Genetics, University of AgricultureFaisalabad, Pakistan; ^18^Centre for Research and Conservation, Royal Zoological Society of AntwerpAntwerp, Belgium; ^19^Department of Chemistry and Bioscience, Aalborg UniversityAalborg, Denmark; ^20^Department of Biology, University of Eastern FinlandKuopio, Finland; ^21^Faculty of Veterinary Medicine, Utrecht UniversityUtrecht, Netherland; ^22^Institute of the Environment and Natural Resources, Makerere UniversityKampala, Uganda; ^23^Animal Breeding and Genomics Centre, Wageningen UniversityWageningen, Netherlands; ^24^School of Planning and Geography, Cardiff UniversityCardiff, UK; ^25^Agroscope, Swiss National Stud FarmAvenches, Switzerland; ^26^TUM School of Management, Technische Universität MünchenMunich, Germany; ^27^Department of Animal Production, Veterinary Faculty, Universidad Complutense de MadridMadrid, Spain; ^28^Croatian Agricultural AgencyZagreb, Croatia; ^29^Department of Biology, Faculty of Science, University of ZagrebZagreb, Croatia; ^30^European Molecular Biology Laboratory, European Bioinformatics Institute, Wellcome Trust Genome CampusHinxton, Cambridge, UK; ^31^School of Agricultural Technology, Walailak UniversityTha Sala, Thailand; ^32^Centre of Research in Animal Health (CIISA) – Faculty of Veterinary Medicine, University of LisbonLisbon, Portugal; ^33^INRA, AgroParisTech, UMR GABIJouy-en-Josas, France; ^34^Department of Biology, University of FribourgFribourg, Switzerland; ^35^Key Laboratory of Animal Ecology and Conservation Biology, Institute of Zoology, Chinese Academy of SciencesBeijing, China; ^36^Cardiff University – Institute of Zoology, Joint Laboratory for Biocomplexity ResearchBeijing, China

**Keywords:** farm animal genetic resources, livestock genetic resources, genomic diversity, livestock population prioritization, effective conservation policy

## Abstract

Livestock conservation practice is changing rapidly in light of policy developments, climate change and diversifying market demands. The last decade has seen a step change in technology and analytical approaches available to define, manage and conserve Farm Animal Genomic Resources (FAnGR). However, these rapid changes pose challenges for FAnGR conservation in terms of technological continuity, analytical capacity and integrative methodologies needed to fully exploit new, multidimensional data. The final conference of the ESF Genomic Resources program aimed to address these interdisciplinary problems in an attempt to contribute to the agenda for research and policy development directions during the coming decade. By 2020, according to the Convention on Biodiversity's Aichi Target 13, signatories should ensure that “…*the genetic diversity of …farmed and domesticated animals and of wild relatives …is maintained, and strategies have been developed and implemented for minimizing genetic erosion and safeguarding their genetic diversity*.” However, the real extent of genetic erosion is very difficult to measure using current data. Therefore, this challenging target demands better coverage, understanding and utilization of genomic and environmental data, the development of optimized ways to integrate these data with social and other sciences and policy analysis to enable more flexible, evidence-based models to underpin FAnGR conservation. At the conference, we attempted to identify the most important problems for effective livestock genomic resource conservation during the next decade. Twenty priority questions were identified that could be broadly categorized into challenges related to methodology, analytical approaches, data management and conservation. It should be acknowledged here that while the focus of our meeting was predominantly around genetics, genomics and animal science, many of the practical challenges facing conservation of genomic resources are societal in origin and are predicated on the value (e.g., socio-economic and cultural) of these resources to farmers, rural communities and society as a whole. The overall conclusion is that despite the fact that the livestock sector has been relatively well-organized in the application of genetic methodologies to date, there is still a large gap between the current state-of-the-art in the use of tools to characterize genomic resources and its application to many non-commercial and local breeds, hampering the consistent utilization of genetic and genomic data as indicators of genetic erosion and diversity. The livestock genomic sector therefore needs to make a concerted effort in the coming decade to enable to the democratization of the powerful tools that are now at its disposal, and to ensure that they are applied in the context of breed conservation as well as development.

## Introduction

Understanding current technical, infrastructural and policy challenges and assessing the likely benefits of overcoming them in the future is essential for any field of scientific endeavor and especially those with clear societal consequences and potential benefits. In this context, the concept of horizon scanning has been developed and applied annually in the field of biodiversity conservation since 2009 (Sutherland and Woodroof, 2009), using a variety of systematic and semi-systematic methods to mine trending issues from web engines and social media and by analyzing focused questionnaires. Similar approaches have also been taken to identify emerging issues in agriculture (Pretty et al., 2010) and related fields such as soil science, food systems and pollination (Dicks et al., 2013; Ingram et al., 2013; Adewopo et al., 2014). Such exercises have identified a number of issues of relevance to the conservation of FAnGR, such as genetic control of invasive species (Sutherland et al., 2014) and sustainable intensification of high yielding agriculture (Sutherland et al., 2015). In 2010, Pretty et al.'s article pinpointing the “*Top 100 questions of importance to the future of global agriculture*” identified genetic issues in crop improvement (e.g., gains in improvement that could result from breeding for stress tolerance) but identified no such pressing agendas for livestock genomic resources. Since Cardellino and Boyazoglu (2009) no attempt has been published to identify research priorities for FAnGR conservation, despite genetic erosion (*sensu* Aichi Target 13) continuing apace (e.g., Berthouly-Salazar et al., 2012; FAO, 2015a) and the step-change that has occurred in molecular breed characterization since the routine implementation of livestock Single Nucleotide Polymorphism (SNP) arrays. To fill this gap, a central activity of the Final Conference of the European Science Foundation's Genomic Resources program, held at Cardiff University June 17th–19th 2014 was to pick out a series of pressing questions that could form part of a research and policy agenda for FAnGR conservation for the next decade. While not following the standard systematic approaches adopted by conventional Horizon Scanning exercises, all 43 attendees of this focused meeting took part in the exercise, including scientists and policy-makers from South and East Asia, North America, Europe and Africa involved in a range of disciplines from genomics to animal breeding, genetic resource management, economic and social sciences and global agricultural policy development.

## Methods and results

During the course of the conference, attendees were asked to contribute up to five questions of highest priority for research, infrastructure and policy development during the coming decade. Eighty-six suggestions were received. The issue identified with highest frequency (18 times) was the need for “next generation phenotyping” (i.e., high-throughput methods to collect and summarize detailed phenotypic data from domestic animals). A summary of the top 20 questions is found in Table 1, a subset of which are presented below (some are amalgamated). All responses were categorized into four major groups, “Methodological Challenges,” “Analytical Challenges,” “Data Management,” and “Conservation Management and Prioritization.” Four working groups were convened to cover these categories and their findings are presented below.

**Table 1:** **Summary of the Top 20 questions in farm animal genomics research identified by the participants of the Cardiff symposium**.

**Question #**	
1. Next generation phenotyping	The mismatch between molecular and phenotypic data has increased dramatically. Which key phenotypic traits should be used as common measures for diversity studies to define breed characteristics in the face of climate change?
2. Genome-wide SNP assays	The identification of common reference genomes and test panels of individuals for SNP array development in less commercial and/or local populations is key. Which strategy shall be used to enable SNP arrays to be developed in a rapid, cost effective and widely applicable manner?
3. Reference genomes	Which common reference genomes and test panels of individuals should be used for array development and diversity studies?
4. E(environment)WAS	How to characterize environmental parameters in extensive production systems?
5. Epigenetics	How can epigenomic information be integrated with phenotypic and genomic data to scrutinize the biological basis for adaptation and plasticity/resilience in livestock populations?
6. Male-mediated genetic diversity	Which methodological approach can be applied to promote reliable assembly of the Y-chromosome, still lacking for many livestock species, as well as to develop polymorphic Y-chromosome markers?
7. Ancient DNA and paleoenvironmental analyses	Which strategies should be followed to collect zooarchaeological specimens from critical geographic sites and promote the analysis of ancient genomes?
8. Conservation of genomic diversity	How to design a management program that evaluates genomic regions for conservation?
9. Polygenic adaptive and economic traits	Haplotypes vs. SNPs: in which situations do one or the other provide a more efficient unit of diversity in QTL regions?
10. Microsatellites (STRs) vs. SNPs	How to integrate data from the STRs and SNPs, and how to manage the transition from STR- to SNP-based characterization of FAnGR?
11. GW diversity statistics	Which combination of parameters will be required to adequately summarize genome diversity?
12. Data management	How can links between major FAnGR databases be promoted to be able to federate resources and act as an educational central point?
13. Data availability	Which format should be used to make NGS, phenotyping and GIS data publicly available, and how can industry contribute toward population and maintenance of such database?
14. Participatory projects	How can participatory projects, including citizen science, for example, the use of smart-phone technologies be encouraged to enable data collection on FAnGR at a large scale?
15. Prioritization for conservation	Why are prioritization methods not being applied by policy makers and managers and is there a lack of dissemination or penetrance?
16. Genomic prioritization	How to implement genomic approaches systematically in conservation prioritization to include genes important in functionally valuable traits?
17. Utilization in practice	How to reconcile the cost of genomic analysis vs. the economic returns on genotyped stock to allow for a wider use of genomic data to assist conservation, production and management of FAnGR? What is the demand and willingness to pay within the sector?
18. Systematic collection	How to ensure that genetic and genomic data are collected sufficiently systematically to be applied to new indicators?
19. Defining goals	Which indicators can be applied to most efficiently monitor genetic trends in domestic populations?
20. Strategic approach	How will the latest advances in ‘omics technology contribute to achieve the ultimate goal of halting the loss of biodiversity of FAnGR?

*Frequencies are not included for each question and the questions are not listed in rank order*.

### Methodological challenges

#### Next generation phenotyping

The need for high-resolution phenotypic data to be collected for in-depth characterization of FAnGR was identified, especially in light of the rapid advances that have been made in molecular breed characterization. Developing methods for phenotypic characterization was also identified by Cardellino and Boyazoglu (2009) following from FAO recommendations (FAO, 2007a) and has clearly remained an under-explored research area. However, with the richness of molecular data increasing dramatically since 2009, the mismatch between molecular and phenotypic data is widening for all except highly commercial transboundary breeds and lines with genomic breeding values. Inherent in high-resolution breed characterization is a need to define key phenotypic traits and characteristics (particularly those potentially involved in local adaptation) based on guidelines that can be used as common measures for such studies with stringent field protocols for their collection. FAO published guidelines on phenotypic characterization (FAO, 2012a). In this way more comparable data can be generated, and breed characterization can have a more functional basis, especially with the urgent need to understand breed characteristics in the face of climate change (Hoffmann, 2010). Also an improved description of the specific production environment and epidemiological history in which populations of a breed are kept would allow better comparison of phenotypes and performances (e.g., FAO, 2009). Since breed characterization can be a costly exercise, especially for remote regions of the world, as many phenotypic traits as possible should be collected following well documented and reproducible procedures, a process that calls for the need for standardized methods to measure/collect data and ultimately for training of people on how to do it. Where possible, data should be made publicly available through a repository such as FAO's global Domestic Animal Diversity Information System DAD-IS (http://dad.fao.org) for comparative purposes. The establishment of a working group to define guidelines, protocols and tools for collecting such data under the auspices of the FAO, International Society for Animal Genetics or the International Committee for Animal Recording (www.icar.org) would accelerate this process.

#### Omics data and association studies

The dramatic acceleration in genome sequencing means that all domesticated species and their few remaining wild relatives will become genome-enabled in the coming decade (e.g., Qiu et al., 2012; Wu et al., 2014). Reference genomes provide the basis for development of genome-wide assays for variation in less commonly farmed and/or more regionally distributed livestock species and populations using SNP arrays, as have been developed and made available for commercial livestock in the past 5 years (e.g., Matukumalli et al., 2009). The choice of SNPs for inclusion in arrays for less commercial populations may be expected to focus on a wider array of traits than for commercial/transboundary breeds, such as those related to local adaptation, disease resistance, drought tolerance and niche product characters, but in practice this could be hampered by a lack of reliable phenotypic data. To enable SNP arrays to be developed in a rapid, cost effective and widely applicable manner, the identification of common reference genomes and test panels of individuals for array development and diversity studies is key. However, it is important to note that with the rapidly falling cost of whole genome resequencing (e.g., Lee et al., 2013; Zhang et al., 2015) using next generation technologies and the availability of even lower cost genotyping by sequencing (GBS: De Donato et al., 2013) being available, the problem of ascertainment bias can be mitigated against since they allow the identification and direct estimation of SNP diversity for FAnGR populations, breeds or species at reasonable prices. Indeed these methods are sufficiently cost-effective now, that they can be in principle used as standard assaying approaches, with a cost in the low tens of dollars for GBS now feasible for analysis of tens of thousands of SNPs.

A major issue identified for genome-wide association studies (GWAS) is experimental design including, but not confined to, sample size considerations (Kadarmideen, 2014) and the availability of different SNP genotyping arrays for some species and their compatibility or lack thereof (Nicolazzi et al., 2015). Characterization of environmental parameters in extensive production systems is another key challenge for GWAS but may be assisted by the application of E(environment)WAS methodologies as applied in humans (e.g., Patel et al., 2010). Additionally, understanding the role of the epigenome and its role in environment-dependent phenotypic diversity and plasticity is becoming an increasing focus in livestock genetics (e.g., Jammes and Renard, 2010; Magee et al., 2011, 2014). Ultimately, the integration of genomic, epigenomic, transcriptomic, and environmental data will be required if meaningful large-scale studies are to be successful in identifying selection and conservation targets in heterogeneous environments (Jones et al., 2013; Wu et al., 2014) and in scrutinizing the biological basis for adaptation, resilience, and even animal improvement.

#### Non-autosomal inheritance

Non-autosomal inheritance (Y-chromosomal, X-chromosomal, and mitochondrial) is a comparatively neglected area of research in livestock conservation. While studies of non-autosomal genetic markers have been extensively used in studies of evolutionary history, both singly and combined (e.g., Götherström et al., 2005; Meadows and Kijas, 2008; Svensson and Götherström, 2008; Pereira et al., 2009; Ramírez et al., 2009; Ginja et al., 2010; Groeneveld et al., 2010), their exploitation in genomic studies has been somewhat overlooked in comparison to autosomal markers in many livestock species. This oversight is surprising given the well-documented links between mitochondrial sequence variation and fitness in human populations (e.g., Wallace, 2005) and the increasingly recognized role that Y-chromosomal variation plays in male fertility in livestock (e.g., Chang et al., 2013; Yue et al., 2014). Technical challenges have long been acknowledged with finding polymorphic markers on the Y-chromosome in mammals and W-chromosome in birds, however such markers, although elusive, have been shown to provide novel insights into livestock diversity when available (e.g., Edwards et al., 2011; Wallner et al., 2013), and should be used as a matter of course to provide a male/female perspective on livestock genomic diversity.

#### Ancient DNA studies

Although firmly established as a major route into a deeper understanding of livestock evolution and diversity (e.g., Larson et al., 2010), ancient DNA (aDNA) studies have been hampered by a number of constraints. These include limited access to samples from geographic areas where (local) domestication may have taken place (e.g., Africa, Near East, Asia, South America), limited data sharing among those groups working on samples from critical sites (but see Arbuckle et al., 2014) and limited success rates, especially for genome-wide studies. Nonetheless, recently developed methodological and bioinformatics tools allowed for increased accuracy in the analysis of high-throughput ancient DNA data and even the characterization of complete genomes of Pleistocene horses (Orlando et al., 2013). Also, alternative sources of material such as parchment are, however, providing promising outcomes (Teasdale et al., 2015). Exciting opportunities have recently been opened up by the discovery of livestock DNA in lake sediment samples in Lake Anterne, Switzerland (Giguet-Covex et al., 2014), which enabled a direct comparison to be made of the paleoenvironment with changes in this environment due to the arrival of farming and domestic livestock, and could be applied to describe historic fluctuations in agricultural intensity and practice and, excitingly, may even allow the possibility of predictive modeling for the presence/absence of suitable agri-habitat under future climate change scenarios.

### Analytical challenges

#### Conservation of genomic diversity

The concept of genome conservation has been discussed extensively in the literature but advances in genome data and technologies only now allow the development of breed management programs able to achieve this aim. For example, Herrero-Medrano et al. (2014), using genome resequencing and SNP arrays discovered almost 100 non-synonymous polymorphic nucleotides nearly fixed in commercial pig breeds but with an alternative allele in non-commercial populations, affecting 65 genes in total. Such genomic polymorphisms could fall into a category of those that “*cannot afford to be lost*” from less commercial local breeds, given their distinctiveness and the value they potentially represent as a genetic resource for alternative selection should the production environment change (Kristensen et al., 2015). However, to design a management program that evaluates genomic regions for conservation, not only do polymorphisms need to be identified, the functional architecture of those genomic regions and the genes they contain needs to be assessed and the interaction among those genes needs to be considered. Recently, a study of chicken breeds examined functional variation in copy number variants (CNV) at over 200 genes overlapping 1000 quantitative trait loci, including some putatively involved in traits such as skin color and skeletal characteristics (Han et al., 2014).

#### Haplotype blocks vs. individual SNPs

Obtaining an accurate description of the genetic polymorphisms explaining a trait of evolutionary, adaptive and/or economic importance is not a trivial task, as traits substantially vary in the number of polymorphisms involved in their phenotype and where these occur across the genome (Goddard and Hayes, 2009; Olson-Manning et al., 2012). For example, many of such traits are polygenic and distributed around the genome, making whole-genome resequencing, and medium and high-density SNP arrays a powerful approach to locating them and elucidating their variation (e.g., Huang et al., 2010). However, for certain linked traits, haplotypes may provide a more efficient unit of assessing diversity in QTL regions than individual SNPs (e.g., Kijas et al., 2013; Bosse et al., 2014a,b; Mokry et al., 2014), reflecting local genomic architecture in a more accurate fashion. Consequently, at the initial stages of studies aiming to identify the genetic basis of phenotypic variation, general genome-wide SNP analyses may be more suitable. It is worth noting, however, that phasing haplotypes in divergent populations lacking complementary pedigree data presents a non-trivial challenge. Haplotype analysis can provide an especially powerful tool to investigate the hybrid origin of domesticated populations. For instance, modern Western commercial pig genomes are a mosaic of Eastern and Western Eurasian biogeographic origin. Admixture mapping allows the “sorting” of haplotype segments for their putative origin. In addition, this strategy has been shown to be powerful to infer selection on specific haplotypes post-hybridization (Bosse et al., 2014a,b).

#### Managing the transition from microsatellite to SNP data

The transition from microsatellite markers to SNPs has happened rapidly in FAnGR for commercial/transboundary breeds due to the availability of relatively inexpensive 50K SNP genotyping arrays for most common livestock species (Matukumalli et al., 2009). However, SNP arrays are not yet affordable tools for much of the world's FAnGR and are not yet available for all species (see above). This therefore raises the immediate problem of how to integrate data from the two marker types and how to manage the transition from microsatellite-based FAnGR characterization (much of which has been carried out using markers recommended by ISAG, FAO, 2011) to SNP-based characterization. One option is to re-genotype many of the breeds that already have microsatellite genotypes with SNPs (Ajmone-Marsan et al., 2014), but this would be expensive and if implemented would raise the question as to whether the new data would again be replaced by a newer technology (e.g., whole-genome resequencing). Pragmatically, it seems that microsatellite data are perfectly adequate for estimating genetic diversity and describing demographic relationships (e.g., Ferrando et al., 2014). However, for cost reasons the full set of microsatellite markers was frequently not applied, especially in developing countries. Also, microsatellite data will not be as efficient for enabling the identification and targeted conservation of genomic regions under selection since data are usually produced with a few tens of quasi-neutral markers (e.g., Herrero-Medrano et al., 2013).

Nevertheless, it is becoming clear that data produced using SNP arrays are more repeatable and do not suffer from scoring differences that have made the combination of microsatellite datasets sometimes problematic and requiring statistical evaluation (Lenstra et al., 2012). Paradoxically, whole genome resequencing may become the most reliable and cost effective way to analyse genomic diversity in the future, even for non-commercial breeds, if the cost comes down by another order of magnitude (as may happen with portable sequencers such as Oxford Nanopore's MiniION system), providing the advantage of no longer needing to use a set of SNP markers ascertained from commercial populations.

#### Genome-wide diversity statistics

The emergence of whole genome sequencing and medium-high density SNP arrays means that summarizing genetic diversity can now be a more nuanced and genomic region-specific exercise. It is well known that ascertainment bias of SNP arrays can strongly underestimate the diversity of the (usually autochthonous and less commercial) breeds not used to design the arrays (Porto Neto and Barendse, 2010). This phenomenon does not impact on whole-genome resequencing as all polymorphisms are captured provided sufficient sequence depth is achieved. A combination of parameters will be required to adequately summarize genome diversity (e.g., heterozygosity and effective population size and inbreeding), as no single all-encompassing statistic to summarize all of a population's genomic diversity and history exists, despite of how tempting it may be to define such statistic (e.g., for policy makers). Effective population size (Ne) estimates can be obtained with as little as a single genome using methods such as the Pairwise Sequential Markovian Coalescent, although these analyses can prove inconclusive if genome coverage is insufficient or if admixture pertains (Li and Durbin, 2011; Orozco-terWengel and Bruford, 2014; Schiffels and Durbin, 2014; Frantz et al., 2015). For recently evolved populations, such as many domestic species, linkage disequilibrium-based (LD) estimates may be more accurate and methods are now emerging to carry out these analysis (e.g., Barbato et al., 2015). Runs of homozygosity (ROH; e.g., Bosse et al., 2012; Scraggs et al., 2014) functions describing the distribution of homozygosity throughout the genome may also serve as a robust genome-scale Ne estimator in the future, although interpretation and scaling depends on the local recombination. ROH are already used as a genomic proxy for inbreeding (e.g., Purfield et al., 2012; Curik et al., 2014), including for specific genome-located traits (Pryce et al., 2014). This approach promises to be an efficient way to avoid the production of offspring homozygous for deleterious alleles at specific genomic regions that are associated with inbreeding depression (Pryce et al., 2014).

### Data management

#### Data accessibility

As also identified by Cardellino and Boyazoglu (2009) there remains a major need to provide much better *links* between the major FAnGR databases, which have largely been set up independently and are breed-focused (Groeneveld et al., 2010). The livestock genomics community needs either to build on an existing platform (such as the ARKDB, http://www.thearkdb.org/arkdb/ and the European Nucleotide Archive, http://www.ebi.ac.uk/ena), that have some level of connectivity, e.g., with Ensembl (http://www.ensembl.org/index.html) or to establish an independent community-based initiative(s) under the form of a user-friendly global web portal and would include web services able to federate resources and act as an *educational* central point. Such resources are already being developed, including the Adaptmap project for goats (http://www.goatadaptmap.org/). Information on livestock related data should be made available and useful recommendations are required to inform stakeholders on how to record data, and where to store what type of information. In particular, it is important to promote within the community of users that raw and meta-data are key components and that they should be made available in public datasets together with elaborated datasets. When there are existing public resources for a given datatype such as those listed above, they should be used for their ability to set standards and centralize data access. For other data types, open digital repositories such as Dryad (http://datadryad.org/), Zenodo (https://zenodo.org/), or figshare (http://figshare.com/) comprise invaluable tools acting as incentives for people to maintain and upgrade their datasets as data can be submitted and authors are provided with a reference which can be cited. This data ecosystem becomes especially important with the myriad of SNP array datasets that are now available and the incompatibility among different versions of these arrays within the same species (Nicolazzi et al., 2015). Moreover, to add value to genetic resources, federating gene bank resources is one step that needs to be completed by explicit connection—through geographical coordinates—with phenotypic data, but also with socio-economic, socio-demographic, climatic, environmental, and policy information. This requires links to existing online digital resources (Joost et al., 2010) that are currently rarely used by the FAnGR community and need to be listed on such a global portal.

#### Data availability

While many genotyping projects on commercial livestock breeds are funded by industry, rendering all except summary data unavailable in many cases, in principle raw data from publicly funded projects should be made publicly available. Indeed, when data are open, it first makes the information more credible, makes data re-usable, and also enables reproducibility an important scientific principle (Ertz et al., 2014). Increasingly, international consortia, such as FAANG on animal functional genomics follow the Toronto protocol and immediately place data in the public domain (http://www.faang.org; The Toronto International Data Release Workshop Authors, 2009; Andersson et al., 2015). A next generation phenotyping database should also be established, including GIS and anonymized farm level data, animal photographs and meta-data—this could partly follow the format of the EU FP5 project Econogene (http://www.econogene.eu) and would be most efficiently linked with FAO's DAD-IS and EFABIS (http://efabis.tzv.fal.de). The ownership and hosting of such a resource would be logistically and financially challenging, and could provide an opportunity for the agri-industry to contribute toward conservation of the genetic resources it has utilized in the past and may need again in the future. This could also be part of the community-based action mentioned above, with many advantages (logistic and funding), but requiring a strong leadership. An approach to data resourcing such has been exemplified with human data by the 1000 Genomes project (http://www.1000genomes.org) and the 1001 Arabidopsis genomes resource (http://1001genomes.org with data being publicly available either immediately or after an agreed embargo period, could be very applicable to livestock studies. For example, the resequencing data from the EU Framework 7 Nextgen project was made available shortly after the project's completion at the European Bioinformatics Institute's FTP site (ftp://ftp.ebi.ac.uk/pub/databases/nextgen/).

#### Participatory projects

Many individuals who are interested in FAnGR are involved in agriculture as smallholders, farmers, breeders, and producers and many of these are not formally involved in breeding programs and livestock conservation, yet maintain an interest through agricultural shows and farmers' markets (e.g., Zimmerer, 2010; Johns et al., 2013). At the same time, the role of participatory approaches and mobile technology potentially enables robust data collection on a previously unimaginable scale (Lisson et al., 2010; Teacher et al., 2013; Sambo et al., 2015). Use of crowdsourcing should therefore be encouraged in FAnGR as should use of smart-phone apps and technologies for photography, data storage and sampling (e.g., “do-forms” http://www.doforms.com). A logical combination of these initiatives lies in the possibility of a livestock community independent initiative, including web services to federate these data sources, to carry out quality control and providing a central access point for data but also information to educate people on how to record FAnGR data. Such approaches could also help in securing funds for projects in FAnGR populations and breeds, which often face the problem of securing funds to carry out this necessary research.

### Conservation, management, and prioritization

#### Is prioritization a priority?

A paradigm within FAnGR for the past 15 years concerns the use of genetic data, alongside other information in prioritization of livestock populations and breeds for conservation (Weitzman, 1992; Simianer et al., 2003; Boettcher et al., 2010; Ginja et al., 2013). However, there is limited evidence that this approach is being applied systematically across countries reporting to the FAO, although the second report on the State of the World's Animal Genetic Resources has documented activities to some extent (FAO, 2015b,c). If, however, prioritization methods are not being applied by managers and policy-makers, the question needs to be asked as to why? A number of explanations may pertain: first, the method(s) may have not gained enough traction with policy makers to ensure its/their implementation, which may indeed be because genomic methods, which have yet to be systematically implemented, will largely supersede the microsatellite-based approaches implemented thus far and enable conservation prioritization to include genes important in functionally valuable traits (e.g., Toro et al., 2014). Furthermore, prioritization on the basis of genetic distances (Weitzman, 1992) is confounded by genetic isolation of breeds (European Cattle Genetic Diversity Consortium, 2006). Second, prioritization may not actually be needed, at least in certain regions, where breed societies are active and all or most of the breeds can be maintained. However, recent animal health emergencies (e.g., outbreaks of transmissible spongiform encephalopathies, TSEs) have cast doubt on this simplistic scenario and required the application of careful genetic management during and after the outbreak. While prioritization may be less of a priority in the world's richest regions, it is not expected to be the case in developing countries, where extinction may take a number of forms, including genetic erosion (e.g., Berthouly-Salazar et al., 2012; FAO, 2015a,b). Finally, the methods developed may not have been applied because policy makers and managers are unaware of their availability, which could be due to a lack of dissemination or penetrance of educational material to the decision makers.

#### Utilization in practice

While research and application of genomic tools in livestock is occurring in many commercial/transboundary breeds (e.g., Pryce et al., 2014; Scraggs et al., 2014), its application in less commercial populations is sporadic and the scientific basis of decisions on management of indigenous livestock, for example in which germplasm to store, assessing the effects of upgrading or evaluating ongoing genetic management is therefore highly variable (e.g., Brown et al., 2014; FAO, 2015b). This points to the reality that genetically-based prioritization is unlikely to be operational in the absence of other considerations, including commercial reality and the ecosystem/production environment (e.g., Sanderson et al., 2013). The use of genomic data to manage FAnGR *within* breeds is however, continuing apace (see above) and can be demonstrated to be assisting conservation, production and management in many cases (e.g., Herrero-Medrano et al., 2014; Scraggs et al., 2014). However, for many breeds the cost of genetic/genomic analysis vs. the potential economic returns on genotyped stock (with a few exceptions such as TSE resistance) makes its application uneconomic, and therefore it is often not applied. It is unlikely that genotyping costs will reach the level of economic viability for many FAnGR, however this assumption should be tested by some targeted research across the sector.

#### Defining goals

The Convention on Biological Diversity's Aichi Target 13, which recommends that: “*strategies have been developed and implemented for minimizing genetic erosion and safeguarding genetic diversity*” is reflected in the Target for Strategic Priority Area 4 of the Global Plan of Action for Animal Genetic Resources (FAO, 2007b). These resource indicators contribute to the measurement of progress toward Aichi Target 13 (FAO, 2012b) and are calculated at national, regional and global levels, based on data entered by National Coordinators for the Management of Animal Genetic Resources^1^ (172 countries had nominated a National Coordinator as of July, 2014) into the Domestic Animal Diversity Information System (DAD-IS). The following indicators have been agreed by the Commission on Genetic Resources for Food and Agriculture:

- the number of locally adapted breeds;
- the proportion of the total population accounted for by locally adapted and exotic breeds; and
- the number of breeds classified as at risk, not at risk and unknown.

The Global Databank for Animal Genetic Resources, the backbone of DAD-IS, enables National Coordinators to enter breed-specific data, including data on the size and structure of breed populations, required to calculate their risk status. FAO produces biannual Status and Trends Reports (FAO, 2015a). For the first report on The State of the Worlds Animal Genetic Resources, a risk status classification based on population size data was used. The (lack of) availability of global data currently makes a more elaborate system involving, for example, molecular diversity indices, population structure/fragmentation, pedigree data, number and size of herds, and geographic distribution inoperable. While genomic methods might help to overcome these data deficiencies, if they are to be applied to livestock conservation, it is important to define the goals of such approaches and how the data could be used to improve or augment the current set of indicators using data that could be collected on trends in effective population size, admixture, inbreeding and genome-wide diversity. The wider application of such data hinges on their applicability to autochtonous, less-commercial breeds. Unfortunately, the data currently provided to FAO does not even allow the reliable calculation of basic trends currently measured via the above indicators (Tittensor et al., 2014; FAO, 2015a), yet the livestock genetics and conservation community possess many of the tools needed to directly evaluate whether signatories to the CBD are “…*minimizing genetic erosion*” and “*safeguarding genetic diversity*” (CBD Target 13). Two key developments are required to enable the current approach to more directly use genetic or genomic data in the future: first, the livestock conservation genetics community must therefore insist that data are collected and analyzed in such a way that results are directly comparable and second, to help develop better indicators applied to monitoring genetic trends in domestic populations.

## Conclusion

Any exercise designed to assess the state-of-the-art in a scientific field only manages to capture a brief moment in time, which is why the Horizon scanning exercises carried out in biodiversity conservation are repeated every year (see Sutherland et al., 2015). Here, we attempted to take a longer-term (decadal) view of genomic resources conservation, and during this period, some major milestones will be passed. Chief among these is the imminent release of the Second Report on the State of the World's Animal Genetic Resources (FAO, 2015b,d) and the Convention on Biological Diversity's 2020 deadline halting the loss of biodiversity Aichi targets. In the context of the dramatic advances in ‘omics technology that are expected during the next decade, the field is expected to move fast. But structural changes in the livestock sector that will bring further erosion during this period are likely to be equally rapid. However, this makes it critically important that a strategic approach is taken to incorporating these technological advances into real world FAnGR conservation. Such an approach has been taken in the past (e.g., with the implementation of approved microsatellite marker sets) and, we would argue, is needed now to ensure that practical conservation of farm animal agricultural biodiversity is not left behind. The FAnGR community therefore needs to make best use of new genomic tools, and at the same time continue and augment its classical phenotyping efforts. Both, genomic and phenotypic tools need to be applied more consistently, at a much wider scale and for more breeds, to describe, utilize and conserve the world's genomic/breed diversity for future generations.

## Disclaimer

The views expressed in this information product are those of the author and do not necessarily reflect the views or policies of FAO.

### Conflict of interest statement

The authors declare that the research was conducted in the absence of any commercial or financial relationships that could be construed as a potential conflict of interest.

## Footnotes

^1^The list of National Coordinators for the Management of Animal Genetic Resources is found at dad.fao.org/cgi-bin/EfabisWeb.cgi?sid=-1,contacts.
